# The Integrated Effects of Brivaracetam, a Selective Analog of Levetiracetam, on Ionic Currents and Neuronal Excitability

**DOI:** 10.3390/biomedicines9040369

**Published:** 2021-04-01

**Authors:** Te-Yu Hung, Sheng-Nan Wu, Chin-Wei Huang

**Affiliations:** 1 Department of Pediatrics, Chi-Mei Medical Center, Tainan 71004, Taiwan; yuchin3344@hotmail.com; 2 Department of Physiology, College of Medicine, National Cheng Kung University, Tainan 70101, Taiwan; 3 Department of Neurology, National Cheng Kung University Hospital, College of Medicine, National Cheng Kung University, Tainan 70101, Taiwan

**Keywords:** brivaracetam, M-type K^+^ current, voltage-gated Na^+^ current, large-conductance Ca^2+^-activated K^+^ channel, neuron, seizure

## Abstract

Brivaracetam (BRV) is recognized as a novel third-generation antiepileptic drug approved for the treatment of epilepsy. Emerging evidence has demonstrated that it has potentially better efficacy and tolerability than its analog, Levetiracetam (LEV). This, however, cannot be explained by their common synaptic vesicle-binding mechanism. Whether BRV can affect different ionic currents and concert these effects to alter neuronal excitability remains unclear. With the aid of patch clamp technology, we found that BRV concentration dependently inhibited the depolarization-induced M-type K^+^ current (*I*_K(M)_), decreased the delayed-rectifier K^+^ current (*I*_K(DR)_), and decreased the hyperpolarization-activated cation current in GH3 neurons. However, it had a concentration-dependent inhibition on voltage-gated Na^+^ current (*I*_Na_). Under an inside-out patch configuration, a bath application of BRV increased the open probability of large-conductance Ca^2+^-activated K^+^ channels. Furthermore, in mHippoE-14 hippocampal neurons, the whole-cell *I*_Na_ was effectively depressed by BRV. In simulated modeling of hippocampal neurons, BRV was observed to reduce the firing of the action potentials (APs) concurrently with decreases in the AP amplitude. In animal models, BRV ameliorated acute seizures in both OD-1 and lithium-pilocarpine epilepsy models. However, LEV had effects in the latter only. Collectively, our study demonstrated BRV’s multiple ionic mechanism in electrically excitable cells and a potential concerted effect on neuronal excitability and hyperexcitability disorders.

## 1. Introduction

Brivaracetam (BRV; Brivact^®^, Brivlera^®^, UCB34714, C_11_H_20_N_2_O_2_), a chemical analog of levetiracetam (LEV), is an orally or intravenously bioavailable racetam derivative with anticonvulsant (antiepileptic) properties that has appeared in a growing number of research papers [1,2,3,4,5,6,7,8,9,10,11,12,13,14,15,16,17,18,19,20,21,22,23,24,25,26,27,28,29,30,31,32,33]. Of note, it has also been recognized to be efficacious in the treatment of epilepsy and status epilepticus [9,17,30,34,35,36,37,38].

BRV has also been reported to attenuate pain behavior in a murine model of neuropathic pain [2,39]. BRV was also previously observed to exert anti-neoplastic effects in glioma cells [40]. It has been demonstrated that BRV can interfere with the functional activities of neurons (e.g., hippocampal neurons) or endocrine cells (e.g., pituitary lactotrophs) by binding with high affinity to the synaptic or endocrine vesicle protein 2A (SV2A) [9,15,39,41,42,43,44,45]. SV2A has been recognized as an important broad marker for neuroendocrine cells [41]. It has been reported to be 10–30-fold more potent than LEV, with high efficacy in a wide range of experimental models of focal and generalized seizures. As a potential medication with significantly high SV2A affinity, it is thus important to investigate its mechanism on neuroendocrine cells and hippocampal neurons. Furthermore, BRV has been demonstrated to have higher potency and efficacy than LEV in experimental models of epilepsy [46]. However, to the best of our knowledge, there is little information available with respect to the effects of BRV on different types of membrane ionic currents in excitatory endocrine cells or neurons or on neuronal excitability, although a previous study showed the ability of BRV to alter the magnitude of voltage-gated Na^+^ current or M-type K^+^ current residing in neurons [42,47]. Furthermore, clinical reports have demonstrated that BRV may be a useful treatment option in patients who have previously failed to respond to or tolerate LEV [48], and it has been reported that BRV has better clinical efficacy and tolerability than LEV [26,28] that cannot be explained simply by the common synaptic vesicle binding property mechanism.

In light of these considerations, we attempted to characterize the effects of BRV on membrane ionic currents (e.g., M-type K^+^ current (*I*_K(M)_), a delayed-rectifier K^+^ current (*I*_K(DR)_), hyperpolarization-activated cation current (*I*_h_), a voltage-gated Na^+^ current (*I*_Na_), and a large-conductance Ca^2+^-activated K^+^ (BK_Ca_) channel) existing in neuroendocrine and hippocampal neurons in order to investigate the integrated effects on neuronal excitability and hyperexcitability using simulation modeling and different animal models of epilepsy.

## 2. Materials and Methods

### 2.1. Chemicals, Drugs, and Solutions Used in This Study

Brivaracetam (BRV, Brivact^®^, Brivlera^®^, UCB34714, ((2S)-2-[(4R)-2-oxo-4-propylpyrrolidin-1-yl]butanamide, C_11_H_20_N_2_O_2_, https://pubchem.ncbi.nlm.nih.gov/compound/Brivaracetam, accessed on 25 March 2021) and LEV (Keppra^®^, (S)-2(2-oxopyrrolidin-1-yl)butanamide) were kindly provided by UCB (Union Chimique Belge) Pharma (PRA Health Sciences, Taipei, Taiwan). GAL-021 and PF1022A were acquired from MedChemExpress (Everything Biotech, New Taipei City, Taiwan); cilobradine was obtained from Cayman (Excel Biomedical, Taipei, Taiwan); 2-chloro-α, α-diphenylbenzeneacetonitrile (TRAM39) was obtained from Tocris (Union Biomed Inc., Taipei, Taiwan), and tefluthrhin, tetraethylammonium chloride and tetrodotoxin were obtained from Sigma-Aldrich (Merck Ltd., Taipei, Taiwan). Unless specified otherwise, culture media, fetal bovine serum, horse serum, L-glutamine, penicillin-streptomycin and trypsin/EDTA were obtained from HyClone^TM^ (Thermo Fisher Scientific; Level Biotech, Tainan, Taiwan), while all other chemicals, including CdCl_2_, CsCl, CsOH, EGTA and HEPES, were of laboratory grade and obtained from standard sources. We used twice-distilled water that had been deionized through a Millipore-Q system (Merck, Ltd., Taipei, Taiwan) in all experiments.

The ionic composition of the bath solution (i.e., HEPES-buffered normal Tyrode’s solution) was: NaCl 136.5 mM, KCl 5.4 mM, CaCl_2_ 1.8 mM, MgCl_2_ 0.53 mM, glucose 5.5 mM, and HEPES 5.5 mM adjusted to pH 7.4 with NaOH. To measure the macroscopic K^+^ currents (e.g., *I*_K(M)_ or *I*_K(DR)_), we filled the recording electrode with a solution: K-aspartate 130 mM, KCl 20 mM, MgCl_2_ 1 mM, Na_2_ATP 3 mM, Na_2_GTP 0.1 mM, EGTA 0.1 mM, and HEPES 5 mM adjusted to pH 7.2 with KOH. To study the BK_Ca_-channel activity measured under an inside-out configuration, the bath solution contained a high K^+^ solution: KCl 130 mM, NaCl 10 mM, MgCl_2_ 3 mM, glucose 6 mM, and HEPES 10 mM titrated to 7.4 with KOH, while the pipette solution contained KCl 145 mM, MgCl_2_ 2 mM, and HEPES 5 mM titrated to 7.4 with KOH. The value of the free Ca^2+^ concentration was estimated in this study, assuming that there was a dissociation constant of 0.1 µM for EGTA and Ca^2+^ (at pH 7.2). For example, to provide 0.1 µM Ca^2+^ in the bath solution, 1 mM EGTA and 0.5 mM CaCl_2_ were added. In this study, we commonly filtered the pipette solutions and culture media with an Acrodisc^®^ syringe filter with a 0.2 µm Super^®^ membrane (Bio-Check Lab., Pall Corp., Taipei, Taiwan).

### 2.2. Cell Culture

The pituitary adenomatous cell line, GH_3_, was acquired from the Bioresource Collection and Research Center (BCRC-60015, http://catalog.bcrc.firdi.org.tw/BcrcContent?bid=60015 (accessed on 26 March 2021), Hsinchu, Taiwan) [49], and the embryonic mouse hippocampal cell line, mHippoE-14, was obtained from Cedarlane CELLutions Biosystems Inc. (Hycell International Co., Taipei, Taiwan) [50,51]. The GH_3_ cells were maintained in Ham’s F-12 medium supplemented with 2.5% fetal bovine serum (*v/v*), 15% horse serum (*v/v*), and 2 mM L-glutamine, and the mHippoE-14 cells were maintained in Dulbecco’s modified Eagle’s medium supplemented with 10% fetal bovine serum (*v/v*) and 2 mM L-glutamine. The cells were grown as a monolayer culture in a humidified environment of 5% CO_2_/95% air at 37 °C. In order to be well differentiated, the GH_3_ cells were transferred to serum- and Ca^2+^-free mediums. Electrical recordings were performed 5 or 6 days after the cells had been cultured to 60–80% confluence.

### 2.3. Electrophysiological Measurements

On the day of the experiments, the cells were dispersed with a 1% trypsin/EDTA solution, and a few drops of cell suspension were quickly transferred to a custom-built recording chamber mounted on the stage of an inverted DM-II microscope (Leica; Major Instruments, Kaohsiung, Taiwan). They were immersed at room temperature (20–25 °C) in normal Tyrode’s solution, the composition of which was provided above. We recorded different types of ionic currents in the whole-cell, cell-attached, or inside-out mode of a standard patch-clamp technique with dynamic adaptive suction (i.e., a decremental change in the suction pressure in response to a progressive increase in the seal resistance), with the aid of either an Axoclamp-2B (Molecular Devices, Sunnyvale, CA, USA) or an RK-400 amplifier (Bio-Logic, Claix, France). Consistent with previous observations [52], the formation of a bleb of membrane lipid in the electrode tip based on microscopic observation of giga-Ω seal formation was observed in our study. The microelectrodes used were prepared from Kimax-51 borosilicate capillaries with a 1.5 mm outer diameter (#34500; Kimble; Dogger, New Taipei City, Taiwan) by using a PP-83 vertical puller (Narishige; Taiwan Instrument, Taipei, Taiwan). Their tip resistance ranged between 3 and 5 MΩ, and they were filled with the various internal solutions described above. During the measurements, the recorded area on the vibration-free table was shielded using a Faraday cage (Scitech, Seoul, Korea). The liquid–liquid junction potential, which commonly appears when the composition of the pipette solution is different from that in the bath, was corrected before the seal formation. The main rationale for the concentrations applied is to use the level which could be clinically achievable and of therapeutic relevance.

### 2.4. Data Recordings

The signals, comprising potential and current tracings, were monitored on an HM-507 oscilloscope (Hameg, East Meadow, NY, USA) and digitally stored online at 10 kHz in an ASUS VivoBook Flip 14 laptop computer (TP412FA-0131A10210U; ASUS, Tainan, Taiwan) equipped with a 12-bit resolution Digidata 1440A interface (Molecular Devices). During the measurements with either analog-to-digital or digital-to-analog conversion, the latter device was controlled using pCLAMP v.10.7 software (Molecular Devices) run on Microsoft Windows 10 (Redmond, WA, USA). A laptop computer was put on the top of an adjustable Cookskin stand (Ningbo, Zheijiang, China) to allow efficient manipulation during the recordings.

### 2.5. Data Analyses

To assess the percentage inhibition of BRV on the *I*_K(M)_ amplitude, each cell was voltage-clamped at −50 mV, and a 1-sec depolarizing pulse to −10 mV was applied. The examined cell was briefly depolarized from −80 to −10 mV. The *I*_K(M)_ and *I*_Na_ amplitudes measured at the beginning or end of the depolarizing pulses in the presence of various concentrations of BRV were compared with the control value (i.e., BRV was not present). The concentration-response data for BRV-induced inhibition of *I*_K(M)_ or peak *I*_Na_ were well fitted with the modified Hill equation (i.e., the 3-parameter logistic equation):

(1) $$\mathrm{Percentage}\;\mathrm{inhibition}\;\left(\%\right)=\frac{E_{max}\times {\left(BRV\right)}^{n_{H}}}{IC_{50}^{n_{H}}+{\left(BRV\right)}^{n_{H}}}$$

where (BRV) represents the BRV concentration; IC_50_ and *n_H_* are the concentrations required for a 50% inhibition and Hill coefficient, respectively, and *E_max_* represents the BRV-mediated maximal block of *I*_K(M)_ or the peak *I*_Na_.

To assess the steady-state inactivation curve of *I*_Na_ measured with or without the addition of BRV, we employed a two-step voltage profile. The relationship between the normalized amplitude of peak *I*_Na_ and the different conditioning potentials was least-squares fitted to a Boltzmann function in the following form:

(2) $$\frac{I}{I_{max}}=\frac{1}{1+exp\left\{\frac{\left(V-V_{1/2}\right)qF}{RT}\right\}}$$

where *V* is the conditioning potential; *V*_1/2_ the potential at which a half-maximal decrease occurs; *I*_max_ is the maximal amplitude of *I*_Na_; *q* is the apparent gating charge in the inactivation curve of the current (i.e., the charge across the membrane’s electrical field between the closed and open conformations); *F* is the Faraday constant; *R* is the universal gas constant; *T* is the absolute temperature, and *RT*/*F* = 25.2 mV.

### 2.6. Single-Channel Analyses

The unitary current events of the digitized BK_Ca_ channels were assessed using a pCLAMP v.10.7 (Molecular Devices). Multi-Gaussian adjustments of the amplitude distributions occurring among the channel events were applied to determine the opening channel event. The functional independence among the channels was validated as the observed stationary probabilities were compared. The open-state probabilities were evaluated using an iterative process to minimize the Χ^2^ value calculated from a sufficient number of independent observations.

### 2.7. Simulation Modeling

To evaluate how BRV could adjust the firing of action potentials (APs), a theoretical model was adapted from a previous work [53]. The model is based largely on the biophysical properties of hippocampal CA1 pyramidal neurons and consists of the delayed-rectifier K^+^ current, the M-type K^+^ current, the transient K^+^ current, the Ca^2+^-activated K^+^ current, the Na^+^ current, and the Ca^2+^ current. A hyperpolarization-activated cation current adapted from a previous work [54] was also included in the model. The conductance values used to solve the set of differential equations are listed in Table 1.

### 2.8. Animal Experiments

All experiments, including the animal procedures, were reviewed and approved by the Institutional Animal Care and Use Committee (IACUC) (Approval No: 110147, Date: 20 February 2021) at National Cheng Kung University, Tainan, Taiwan. Adult Sprague-Dawley male rats weighing 180 to 200 g were purchased from National Cheng Kung University. They were housed in the university’s Animal Center and allowed free access to water and a pelleted rodent diet (Richmond Standard; PMI Feeds, St Louis, MO, USA). All efforts were made to minimize the number of rats used. The animals were divided into two groups (OD-1 and lithium-pilocarpine). We previously characterized the OD-1 group and its effect as a novel animal model [54].

The OD-1 group was stereotactically injected with 5 ng OD-1 (12 mouse LD50) in the hippocampus (coordinates 4.1 mm caudal, 3.9 mm lateral to the bregma and 3.8 mm below the cortical surface) under general anesthesia (zoletil, 0.1 mL/100 g, intraperitoneal injection (ip)) and analgesia (nalbuphine, 5 mg/kg, ip). After recovery from anesthesia (24 h after injection), the OD-1 group was then divided into three groups given normal saline (NS, 0.1 mL/100g, an intraperitoneal injection (ip)), LEV (500 mg/kg, ip), or BRV (100 mg/kg, ip). The acute seizures were carefully monitored and scored in the following eight hours.

The rats in the pilocaprine group were divided into three groups given NS (0.1 mL/100 g, ip), LEV (500 mg/kg, ip), or BRV (100 mg/kg, ip) 20 min before the pilocarpine injection. The lithium-pilocarpine group was injected with lithium chloride (3 meq/kg; ip) and methylscopolamine (25 mg/kg; subcutaneous (sc)) before they were subjected to pilocarpine (60 mg/kg; subcutaneous injection (sc))-induced seizures.

In both of the OD-1 and lithium-pilocarpine groups, the seizure characteristics of the rats during acute seizures were similar to those reported elsewhere [51,55,56]. The seizures were scored using the Racine scale [56]. The rats were given zoletil (50 mg/kg, ip), xylazine (20 mg, ip), and atropine (0.2 mg/kg, sc) to diminish the seizures if their status epilepticus lasted for 20 min [51,55,56,57,58]. Mortality was calculated during the first 24 h after seizure onset. All rats were continuously monitored for the first 24 h after they achieved status epilepticus by two experienced research assistants. The rats were given supportive care: body temperature maintenance with a resistive heating system, food, and adequate hydration with normal saline (0.9% *w/v* of NaCl, 308 mOsm/L). Any animals showing intense signs of acute respiratory distress were immediately euthanized by overdosing with sodium pentobarbital (150 mg/kg, ip).

In OD-1 model, we evaluated the parameter severe seizures as an indicator of higher neuronal excitotoxicity. In pilocarpine model, the latency to acute stage 3 seizures, number of rats with severe seizures and acute mortality were used to evaluate the neuronal excitotoxicity.

### 2.9. Statistical Analyses

Linear or nonlinear curve-fitting (e.g., exponential curve or sigmoidal Hill and Boltzmann equations) to any given data sets was undertaken, with the goodness of fit assessed using either Microsoft Excel^®^ v.2016 (Redmond, WA, USA) or OriginPro v.2016 (OriginLab; Schmidt, Taipei, Taiwan). Values are provided as means ± SEM with sample sizes (*n*), which indicate the number of GH_3_ cells or mHippoE-14 neurons from which the experimental data were collected. The Student’s *t*-test and a one-way analysis of variance (ANOVA) followed by post-hoc Fisher’s least-significant difference test for multiple comparisons were performed. However, the data were examined using the nonparametric Kruskal–Wallis test, subject to possible violations in the normality underlying the ANOVA. Differences were considered statistically significant when the *p*-value was less than 0.05.

## 3. Results

### 3.1. Inhibitory Effect of BRV on the Amplitude of M-Type K^+^ Current (I_K(M)_)

**Figure 1:**
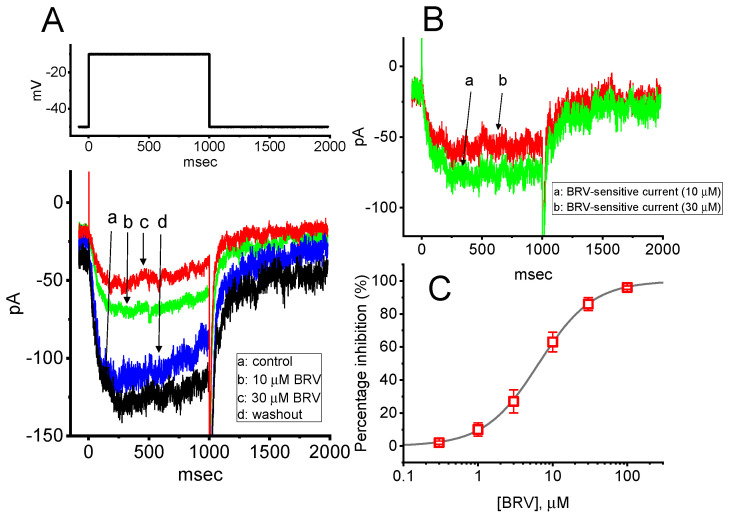
Inhibitory effect of BRV on M-type K^+^ current (*I*_K(M)_) in GH_3_ cells. In these experiments, cells were immersed in high-K^+^, Ca^2+^-free solution, and the recording electrode was filled with the K^+^-containing solution comprising: K-aspartate 130 mM, KCl 20 mM, MgCl_2_ 1 mM, Na_2_ATP 3 mM, Na_2_GTP 0.1 mM, EGTA 0.1 mM, and HEPES 5 mM adjusted to pH 7.2 with KOH; (**A**) Representative *I*_K(M)_ traces evoked by a 1 s depolarizing pulse from −50 to −10 mV (indicated in the upper part of the figure). (a): control (i.e., BRV was not present); (b): 10 µM BRV; (c): 30 µM BRV; (d): washout of BRV. It should be noted that the trajectories of the deactivating currents were partly cut off for the purpose of illustration; (**B**) BRV-sensitive inward current (a–b (green trace) or a–c (red trace) in panel (**A**)) (a): BRV-sensitive current at 10 µM BRV; (b): BRV-sensitive current at 30 µM BRV; (**C**) Concentration-dependent inhibition of BRV on *I*_K(M)_ amplitude measured from GH_3_ cells (mean ± SEM; *n* = 8). Current amplitude obtained in the absence and presence of different BRV concentrations (0.3–100 µM) was taken at the endpoint of depolarization pulses ranging from −50 to −10 mV. A smooth, continuous line was obtained by fitting the experimental results to the modified Hill equation (as elaborated in the Materials and Methods section). The vertical broken line points out the IC_50_ value (i.e., 6.5 µM) needed for BRV-mediated inhibition of *I*_K(M)_ in these cells.

The initial stage of the experiments was undertaken to evaluate the effect of BRV on the *I*_K(M)_ inherently existing in the GH_3_ cells. We bathed the cells in a high-K^+^, Ca^2+^-free solution, and, during the recordings, we backfilled the pipette using a K^+^-containing solution comprising: K-aspartate 130 mM, KCl 20 mM, MgCl_2_ 1 mM, Na_2_ATP 3 mM, Na_2_GTP 0.1 mM, EGTA 0.1 mM, and HEPES 5 mM adjusted to pH 7.2 with KOH. When the whole-cell mode was firmly established, we voltage-clamped the cell at −50 mV and thereafter applied a 1 s depolarizing pulse ranging from −50 to −10 mV. As expected, an inward current with a slowly activating time course was evoked. This current has previously been identified to be *I*_K(M)_ [59,60,61]. It was clearly observed that, as the cells were exposed to different concentrations of BRV, the amplitude of *I*_K(M)_ progressively decreased (Figure 1A). For example, the addition of 10 µM BRV decreased the *I*_K(M)_ amplitude from 78 ± 8 to 42 ± 4 pA (*n* = 8, *p* < 0.05). After it was removed, the current amplitude returned to 74 ± 6 pA (*n* = 7, *p* < 0.05). The activation time course in the presence of BRV (10 µM) slowed, as evidenced by a significant prolongation in the activation time constant (τ_act_) of the current from 92 ± 11 to 122 ± 14 msec (*n* = 8, *p* < 0.05). Moreover, when the differences in the current traces between the absence and presence of 10 or 30 µM BRV were taken, the net change in the membrane currents, i.e., the BRV-sensitive component, was obtained (Figure 1B). These BRV-sensitive inward currents exhibited time-dependent activation and deactivation. Figure 1C shows that cell exposure to BRV can result in a concentration-dependent decrease in the amplitude of *I*_K(M)_ elicited in response to 1 s step depolarization. The IC_50_ value needed for BRV-perturbed inhibition of *I*_K(M)_ was estimated to be 6.5 µM. Therefore, BRV is capable of producing a depressant action on depolarization-induced *I*_K(M)_.

### 3.2. Mild Inhibition of BRV on Delayed-Rectifier K^+^ Current (I_K(DR)_)

**Figure 2:**
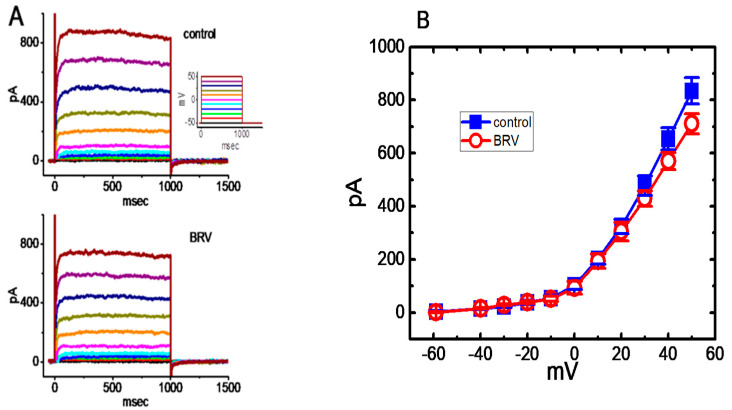
Mild inhibition of BRV on delayed-rectifier K^+^ current (*I*_K(DR)_) in GH_3_ cells. In these experiments, the cells were bathed in Ca^2+^-free Tyrode’s solution containing 1 µM tetrodotoxin and 0.5 mM CdCl_2_, where the recording electrode was filled with a K^+^-containing solution comprising: K-aspartate 130 mM, KCl 20 mM, MgCl_2_ 1 mM, Na_2_ATP 3 mM, Na_2_GTP 0.1 mM, EGTA 0.1 mM, and HEPES 5 mM adjusted to pH 7.2 with KOH. (**A**) Representative *I*_K(DR)_ traces obtained in the absence (upper) and presence of 10 µM BRV. The inset in the upper part of the figure indicates the voltage profile used; (**B**) Comparison of the mean current-voltage (IV) of *I*_K(DR)_ without (○) or with the addition (■) of 10 µM BRV (mean ± SEM; *n* = 8). The current amplitude was measured at the end of each voltage command pulse. It should be noted that BRV (30 µM) mildly inhibited the *I*_K(DR)_ amplitude at +50 mV.

The question as to whether BRV affects other types of K^+^ currents (e.g., *I*_K(DR)_) was raised. Previous reports have shown the ability of LEV to modify the amplitude and gating of *I*_K(DR)_ [62]. The subsequent experiments were therefore performed to evaluate whether *I*_K(DR)_ could be modified in the presence of BRV. To elicit a family of *I*_K(DR)_ (Figure 2A), cells were immersed in Ca^2+^-free Tyrode’s solution containing 1 µM tetrodotoxin and 0.5 mM CdCl_2_, and the recording electrode was filled with K^+^-containing solution. During the measurements, the examined cell was maintained at −50 mV, and various voltage steps ranging between −60 and +50 mV at intervals of 10 mV were applied to evoke *I*_K(DR)_ [61,62]. Under these conditions, BRV at a concentration of 3 µM was not found to have any effect on *I*_K(DR)_, as measured throughout the entire voltage-clamp step. For example, as the cells were depolarized from −50 to +50 mV, the *I*_K(DR)_ amplitude in the absence and presence of 3 µM BRV did not differ significantly (822 ± 42 pA (control) vs. 821 ± 22 pA (the presence of BRV); *n* = 8, *p* > 0.05). However, when the cells were exposed to 10 µM BRV, the amplitude of *I*_K(DR)_ slightly, but significantly, decreased (Figure 2A,B). For example, at +50 mV, the current amplitude significantly declined from 834 ± 49 to 711 ± 38 pA (*n* = 8, *p* < 0.05) during cell exposure to 10 µM BRV. The current-voltage (I-V) relationship to *I*_K(DR)_ collected with or without the addition of 10 µM BRV is illustrated in Figure 2B. It could be observed that, unlike *I*_K(M)_, *I*_K(DR)_ tends to be less subject to being altered by BRV.

### 3.3. Mild Inhibitory Effect on Hyperpolarization-Activated Cation Current (I_h_) Caused by BRV

**Figure 3:**
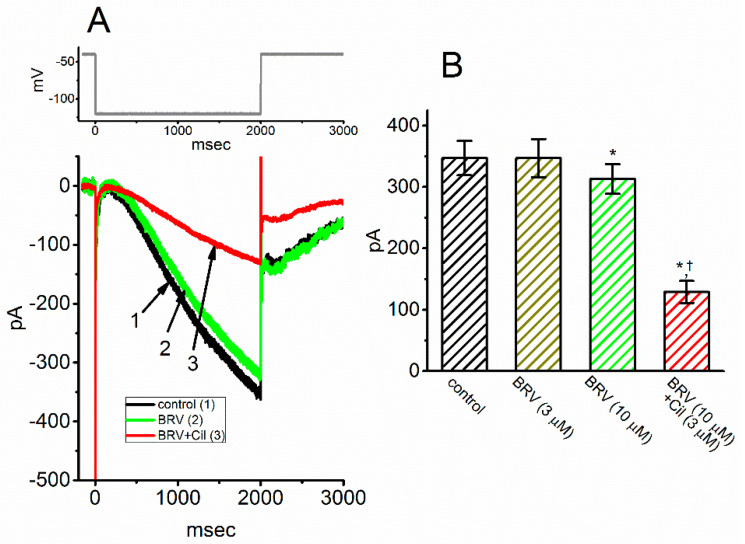
Mild inhibition of BRV on hyperpolarization-activated cationic current (*I*_h_) in GH_3_ cells. The experiments were conducted in cells immersed in Ca^2+^-free Tyrode’s solution containing 1 µM tetrodotoxin, where the pipette was filled with a K^+^-containing solution comprising K-aspartate 130 mM, KCl 20 mM, MgCl_2_ 1 mM, Na_2_ATP 3 mM, Na_2_GTP 0.1 mM, EGTA 0.1 mM, and HEPES 5 mM adjusted to pH 7.2 with KOH. (**A**) Representative *I*_h_ traces activated by a 2 s hyperpolarizing voltage pulse ranging from −40 to −120 mV (indicated in the upper part of the figure). (1): control, (2): 10 µM BRV, and (3): 10 µM BRV plus 3 µM cilobradine (Cil); (**B**) Summary bar graph showing the effects of BRV and BRV plus cilobradine (Cil) in hyperpolarization-activated *I*_h_ (mean ± SEM; *n* = 8). The current amplitude was measured at the endpoint of a 2 s hyperpolarizing pulse ranging from −40 to −120 mV. * indicates significantly different from control (*p* < 0.05) and † indicates signficantly different from BRV (10 mM) alone group (*p* < 0.05).

We further examined whether BRV could produce any modifications on hyperpolarization-induced *I*_h_. The experiments were conducted in cells bathed in Ca^2+^-free Tyrode’s solution containing 1 µM tetrodotoxin, and the recording electrode was filled with a K^+^-containing solution. As depicted in Figure 3A, the 2 s long hyperpolarizing command voltages ranging from −40 to −120 mV could readily evoke an inward current with slowly activating and deactivating time courses in response to such sustained hyperpolarization. This type of ionic current has been previously identified to be *I*_h_ [49,63,64]. When the cells were exposed to BRV (3 µM), the *I*_h_ amplitude was unaffected. However, BRV at a concentration of 10 µM resulted in a lessening in the *I*_h_ amplitude from 347 ± 28 to 313 ± 24 pA (*n* = 7, *p* < 0.05). Additionally, in the continued presence of 10 µM BRV, subsequent addition of 3 µM cilobradine was observed to lessen the current amplitude further, as demonstrated by a significant reduction in current amplitude to 129 ± 18 pA (*n* = 7, *p* < 0.05) (Figure 3B). Cilobradine was previously used to effectively suppress *I*_h_ [65].

### 3.4. Effect of BRV on Voltage-Gated Na^+^ Current (I_Na_)

**Figure 4:**
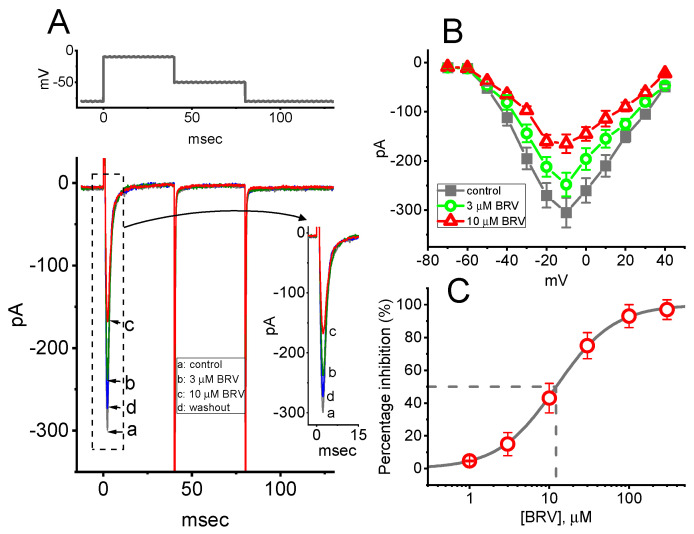
Inhibitory effects of BRV on voltage-gated Na^+^ current (*I*_Na_) in GH_3_ cells. The cells were bathed in Ca^2+^-free Tyrode’s solution containing 10 mM tetraethylammonium chloride, where the recording electrode was filled with a Cs^+^-containing solution. (**A**) Representative *I*_Na_ traces activated by rapid membrane depolarization (indicated in the upper part of the figure). Inset shows an expanded record from the dashed box. (a): control (i.e., BRV not present), (b): 3 µM BRV, (c): 10 µM BRV and (d): washout of BRV. (**B**) Mean I-V relationships of peak *I*_Na_ obtained in the absence (●) and in the presence of 3 µM BRV (○) and 10 µM BRV (△) (mean ± SEM; *n* = 7–8). The current amplitude was measured at the beginning of each brief depolarization. It should be noted that cell exposure to BRV depressed the amplitude of the peak *I*_Na_; however, the overall *I-V* relationship with the current was not altered. Furthermore, the activation and inactivation time courses of the peak *I*_Na_ were clearly modified in the presence of BRV. (**C**) Concentration-dependent inhibition of peak *I*_Na_ caused by different BRV concentrations (mean ± SEM; *n* = 7). Peak *I*_Na_ was activated by rapid depolarization from −80 to −10 mV. The vertical broken line was placed on the IC_50_ value of BRV used to show the inhibition of peak *I*_Na_ amplitude in response to the depolarizing command voltage.

In the next set of experiments, the *I*_Na_ was examined in response to a short depolarizing command voltage to determine whether BRV could modify it. Cells were bathed in Ca^2+^-free Tyrode’s solution containing 10 mM tetraethylammonium chloride, and the electrode was filled with a Cs^+^-containing solution comprising: Cs-aspartate 130 mM, CsCl 20 mM, MgCl_2_ 1 mM, Na_2_ATP 3 mM, Na_2_GTP 0.1 mM, EGTA 0.1 mM, and HEPES 5 mM adjusted to pH 7.2 with CsOH. As shown in Figure 4A, when the examined cell was rapidly depolarized from −80 to −10 mV, *I*_Na_ with both a rapid activation and inactivation time course was robustly evoked. As the cells were exposed to BRV, the peak amplitude of *I*_Na_ in response to a brief depolarizing command voltage progressively declined. For example, BRV (10 µM) resulted in an evident reduction in peak *I*_Na_ from 298 ± 19 to 153 ± 11 pA (*n* = 8, *p* < 0.05). After the washout of BRV, the current amplitude returned to 291 ± 17 pA (*n* = 8, *p* < 0.05). However, neither activation, inactivation, nor deactivation of the time course of *I*_Na_ in response to brief step depolarization was measurably perturbed in the presence of BRV (3 or 10 µM). µ illustrates the mean *I-V* relationships of peak *I*_Na_ in the absence and presence of 3 or 10 µM BRV. It was observed that the overall *I-V* relationship to peak *I*_Na_ in these cells was not altered during exposure to BRV, in spite of an obvious lessening in peak *I*_Na_. The concentration-dependent inhibitory effect of BRV on peak *I*_Na_ amplitude was then determined, as illustrated in Figure 4C. According to the modified Hill equation elaborated in the Materials and Methods Section, the IC_50_ values for BRV-induced inhibition of peak *I*_Na_ measured at the start of the depolarizing command voltage was found to be 12.2 µM, and BRV at a concentration of 300 µM almost completely eliminate the current amplitude.

### 3.5. Steady-State Inactivation Curve of Peak I_Na_ Taken with or without Addition of BRV

**Figure 5:**
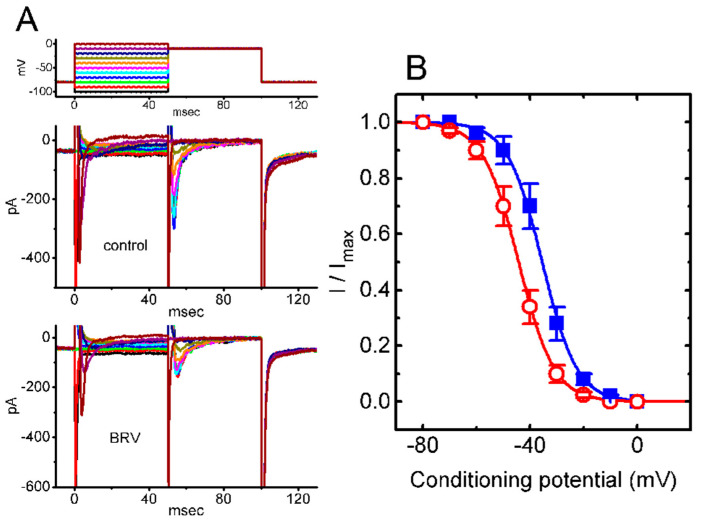
BRV-induced modifications to the steady-state inactivation curve of peak *I*_Na_ recorded from GH_3_ cells. In this set of experiments, the cells were immersed in a Ca^2+^-free Tyrode’s solution containing 10 mM tetraethylammonium chloride, where the pipette was filled with Cs^+^-containing solution. (**A**) Representative *I*_Na_ traces obtained without (upper view) and with addition of 10 µM BRV (lower view). The uppermost part indicates the voltage profile used, where the different colors correspond to the different command voltages; (**B**) Steady-state inactivation curve of peak *I*_Na_ in the absence (■) and presence (○) of 10 µM BRV (mean ± SEM; *n* = 7). It should be noted that a significant leftward (i.e., hyperpolarizing) shift along the voltage axis in the inactivation curve of the current was detected in the presence of 10 µM BRV), while the slope (i.e., gating charge [*q*]) of the curve remained unaffected.

The following experiments were undertaken to investigate the effects of BRV on the steady-state inactivation of *I*_Na_. In this set of whole-cell recordings, a two-pulse protocol was used (Figure 5A), and the inactivation parameters of peak *I*_Na_ were then estimated in the absence or presence of 10 µM BRV. The normalized amplitude of peak *I*_Na_ (i.e., *I/I_max_*) versus the conditioning potential was then derived, as presented in Figure 5B. Thereafter, the experimental data were least-squares fitted (indicated in the continuous smooth line) to a Boltzmann function elaborated in the Materials and Methods Section (Figure 5B). Based on the experimental observations, in the control (i.e., BRV was not present), *V*_1/2_ = −35.5 ± 2.9 mV, *q* = 3.8 ± 0.2 *e* (*n* = 7), whereas in the presence of 10 µM BRV, *V*_1/2_ = −44.3 ± 3.1 mV, *q* = 3.7 ± 0.2 *e* (*n* = 7). Noticeably, as cells were exposed to 10 µM BRV, the midpoint of the steady-state inactivation curve was shifted in a hyperpolarizing direction by approximately 9 mV, regardless of its inability to alter the estimated gating charge (i.e., *q* value) of the curve. The results from these experiments made it possible to indicate that exposure to BRV is capable of altering the inactivation curve of *I*_Na_.

### 3.6. Stimulatory Effect of BRV on the Activity of Large-Conductance Ca^2+^-Activated K^+^ (BK_Ca_) Channels

**Figure 6:**
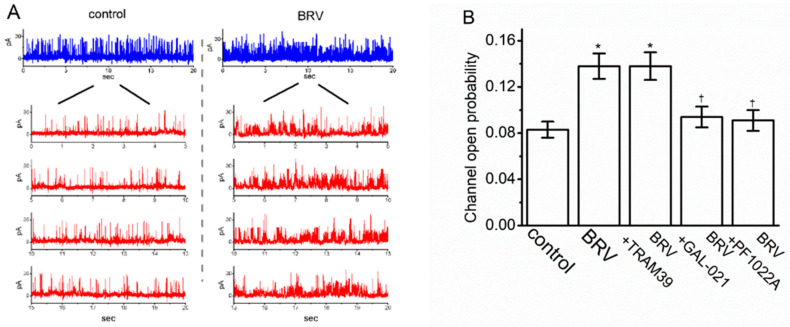
Stimulatory effect of BRV on the activity of the large-conductance Ca^2+^-activated K^+^ (BK_Ca_) channels in GH_3_ cells. In this set of inside-out current recordings, the cells were immersed in a high-K^+^ solution containing 1 µM Ca^2+^, where the holding potential was clamped at +60 mV. (**A**) Representative BK_Ca_-channel traces (blue color) obtained in the control (i.e., BRV was not present) (left side of the figure) and after the bath addition of 10 µM of BRV (right side of the figure). It should be noted that the upward deflection shows the channel opening event. The lower part (red color) of the figure shows the expanded records taken from the uppermost part of the figure; (**B**) Summary bar graph showing the open-state likelihood of the presence of BK_Ca_ channels in the presence of BRV (10 µM), BRV (10 µM) plus TRAM39 (3 µM), BRV (10 µM) plus GAL-021 (3 µM), and BRV (10 µM) plus PF1022A (3 µM) (mean ± SEM; *n* = 7–8). The channel activity was measured when the membrane patch was maintained at +60 mV. * Significantly different from the control (*p* < 0.05) and ^Ɨ^ significantly different from the 10 µM BRV-alone group (*p* < 0.05).

Next, an effort was made to explore whether the presence of BRV could produce any perturbations on the probability of which BK_Ca_ channels would be actively open. Cells were bathed in high-K^+^ solution (i.e., 130 mM K^+^) that contained 1 µM Ca^2+^, and the recording pipette was filled with the K^+^-containing solution. When the inside-out configuration was firmly established, the excised membrane was voltage-clamped at +60 mV. As depicted in Figure 6A, the activity of the BK_Ca_ channels was drastically increased as the intracellular leaflet of the detached patch was exposed to 10 µM of BRV. For example, the presence of resulted in a clear increase in the channel open-state probability from 0.083 ± 0.007 to 0.138 ± 0.011 (*n* = 8, *p* < 0.05); however, it was observed that 10 µM of BRV was unable to alter the single-channel amplitude. When the BRV was washed out, the channel activity returned to 0.094 ± 0.008 (*n* = 6, *p* < 0.05). Alternatively, in the continued presence of BRV, the subsequent addition of GAL-021 or PF1022A reversed the BRV-mediated decrease in BK_Ca_-channel activity; however, further application of TRAM39 failed to exert any effects (Figure 6B). TRAM39 was previously reported to be an inhibitor of intermediate-conductance Ca^2+^-activated K^+^ channels [66], whereas GAL-021 or PF1022A alone was shown to effectively suppress BK_Ca_-channel activity [64,67].

### 3.7. Inhibitory Effect of BRV on I_Na_ in Hippocampal Neurons

**Figure 7:**
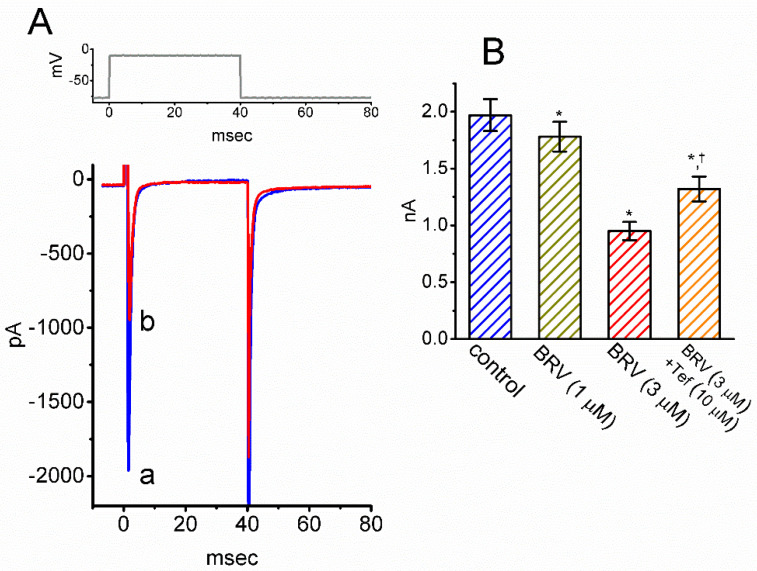
Inhibitory effect of BRV on *I*_Na_ in mHippoE-14 hippocampal neurons. (**A**) Representative *I*_Na_ traces obtained in the absence (a, blue color) and presence (b, red color) of 3 µM BRV. The upper part is the voltage protocol delivered; (**B**) Summary bar graph showing the effects of BRV and BRV plus tefluthrin (10 µM) on the peak amplitude of *I*_Na_ in these cells (mean ± SEM; *n* = 8). * Significantly different from control (*p* < 0.05) and ^Ɨ^ significantly different from 3 µM BRV-alone group (*p* < 0.05).

BRV has been reported to produce significant changes in the functional activities of neurons or neural networks that include the hippocampus [12,29,39,43,47]. To investigate this result, experiments were undertaken to determine whether the *I*_Na_ residing in mHippoE-14 cells [68] could be subject to any modifications by BRV. Whole-cell current recordings were conducted in cells that were bathed in Ca^2+^-free Tyrode’s solution, and the electrode was filled with a Cs^+^-containing solution. As the cells were exposed to different concentrations of BRV, the amplitude of the peak *I*_Na_ was activated in response to a brief depolarizing pulse (Figure 7A,B). For example, BRV at a concentration of 3 µM lessened the amplitude of peak *I*_Na_ from 1.97 ± 0.14 to 0.95 ± 0.08 nA (*n* = 8, *p* < 0.05). Furthermore, when 3 µM BRV was continually present, further addition of tefluthrin (10 µM) was found to reverse BRV-mediated inhibition of the *I*_Na_ amplitude, as evidenced by a decrease in the peak *I*_Na_ amplitude to 1.32 ± 0.11 nA (*n* = 8, *p* < 0.05) (Figure 7B). Tefluthrin, a type-I pyrethroid insecticide, has been previously demonstrated to activate *I*_Na_ [69]. Therefore, in keeping with the observations in the GH_3_ cells discussed above, the presence of BRV was shown to produce a depressant action on the *I*_Na_ observed in mHippoE-14 cells; however, the activation and inactivation time courses of the current were not modified in its presence.

### 3.8. Effect of BRV on BK_Ca_-Channel Activity Recorded from mHippoE-14 Hippocampal Neurons

**Figure 8:**
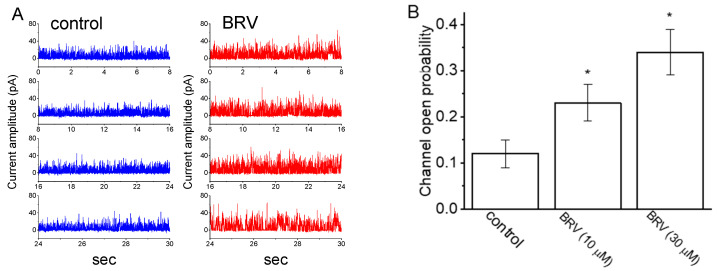
Stimulatory effects of BRV on the activity of BK_Ca_ channels identified in mouse mHippoE-14 hippocampal neurons. In these experiments, cells were bathed in high K^+^ solution containing 1 µM of Ca^2+^; inside-out current recordings were performed, and channel activity was measured at a holding potential of +60 mV. (**A**) Representative channel current traces obtained in the control period (left, blue color) and after bath addition of 10 mM BRV (red color). Channel-opening event is denoted as an upward deflection (i.e., outward current); (**B**) Summary bar graph showing effects of BRV on the probability of channel opening during exposure to 10 or 30 mM of BRV (mean ± SEM; *n* = 8). * Significantly different from control (*p* < 0.05).

A further investigation of the effects of BRV on the activity of BK_Ca_ channels in mHippoE-14 cells was carried out. Under inside-out current recordings, cells were bathed in a high-K^+^ solution containing 1 µM Ca^2+^, and the potential was voltage-clamped at +60 mV. As demonstrated in Figure 8A,B, the probability that BK_Ca_ channels would be open was elevated when the excised patch was exposed to 10 or 30 µM of BRV. Figure 8B provides a summary bar graph showing the stimulatory effects of BRV on the channel opening probability in these cells. Therefore, the presence of BRV had a stimulatory effect on the BK_Ca_-channel activity in these cells.

### 3.9. Simulated Firing of Action Potentials (APs) in Modeled Neurons Used to Mimic the Effect of BRV

**Figure 9:**
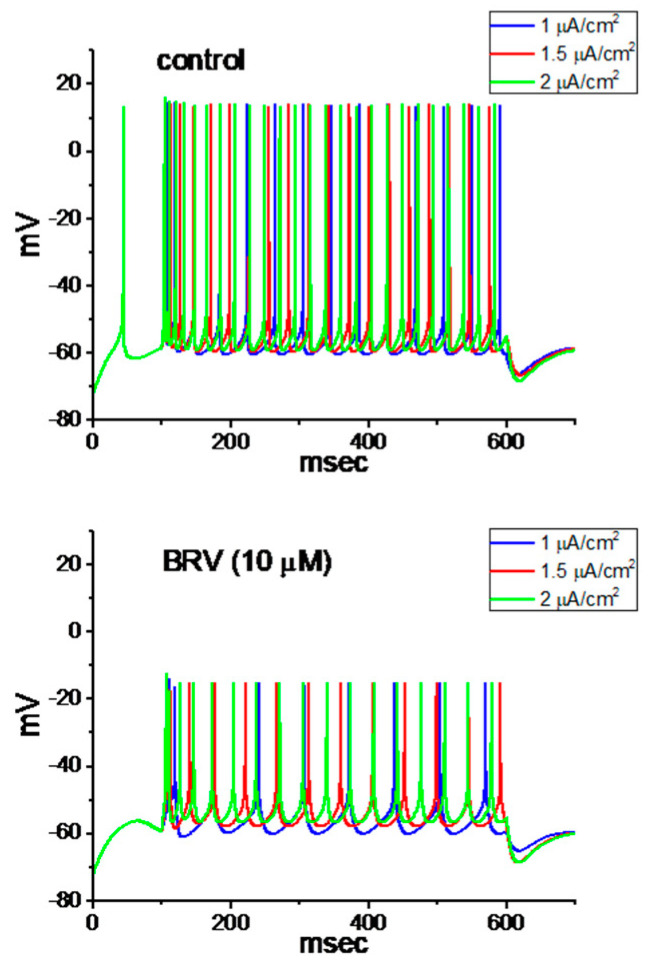
Simulated firing of action potentials (APs) generated from the modeled neuron to mimic the effects of BRV. AP traces in the upper and lower part of the figure indicate those theoretically simulated to mimic the control condition in which BRV was not present and the experimental condition in the presence of BRV (10 µM), respectively. The AP firing in each panel was evoked by a 500 ms current stimulus at a strength of 1, 1.5 and 2 µA/cm^2^. The values used for this numerical simulation are illustrated in Table 1.

For further evaluation of the effect of BRV on neuronal excitability, we explored how the firing of APs in a modeled neuron can be adjusted by adding BRV. The descriptions of this modeled neuron were detailed previously [53], and the formula for a hyperpolarization-activated cation current was incorporated into the model [54]. The parameters used in this work are illustrated in Table 1. In attempts to study AP firing, a long depolarizing current with 1, 1.5, or 2 mA/cm^2^ was applied to this modeled neuron. It is clear from these simulations that, with different current stimulus strengths, the presence of 10 µM of BRV (i.e., with arbitrary changes in the different types of ionic currents demonstrated above) led to decreases in the AP firing frequency as well as the AP amplitude of the modeled neuron (Figure 9).

### 3.10. Effects of BRV versus LEV on Acute Seizures in Different Animal Models

**Figure 10:**
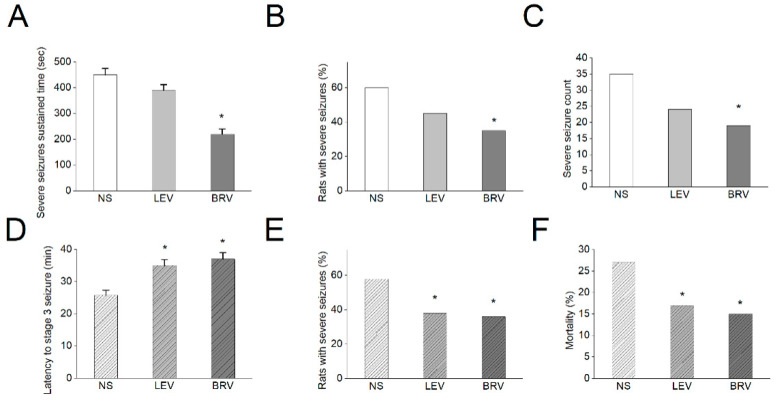
Effects of BRV versus LEV on acute seizures in OD-1 and lithium-pilocarpine animal models. (**A**–**C**) In the OD-1 model, BRV had a significant effect on the sustained time of severe seizures (stage 4 and above) compared to the control group. The BRV group also had a low number of rats with severe seizures and a lower severe seizure count, as compared to the control group (* *p* < 0.05, *n* = 7 in each group); (**D**–**F**) In the lithium-pilocarpine-induced epilepsy model, compared to the control group, both LEV and BRV had significant effects on the latency of stage 3 seizures, the number of rats with severe seizures, and mortality (* *p* < 0.05, *n* = 7 in each group). The data were analyzed using an ANOVA followed by Fisher’s least significant difference tests.

In a final series of studies, we evaluated the effects of BRV versus LEV on acute seizures in different animal models, including a sodium channel agonism-based OD-1 model [55] and the well-established lithium-pilocarpine-induced epilepsy model. We have recent characterized OD-1, a scorpion toxin, which could produce a concentration-, time-, and state-dependent rise in the peak amplitude of *I*_Na_, shifting the *I*_Na_ inactivation curve to a less negative potential and increasing the frequency of spontaneous action currents. It could generate a significantly higher frequency of spontaneous seizures and epileptiform discharges compared with lithium-pilocarpine- or kainic acid-induced epilepsy, with comparable pathological changes [55]. We found in the OD-1 model that BRV had significant effects on the sustained time of severe seizures (stage 4 and above, seconds) (NS: 450 ± 25, LEV: 390 ± 22, BRV: 220 ± 20) (*p* < 0.05) compared to the control group (Figure 10A). The BRV group also had a significantly lower number of animals with severe seizures (NS: 60%, LEV: 45%, BRV: 35%) (*p* < 0.05) and severe seizure counts within the 8 h study duration (NS: 35, LEV: 24, BRV: 19) (*p* < 0.05) as compared to the control group (Figure 10B,C). In comparison, in the lithium-pilocarpine-induced epilepsy model, compared to the control group, both LEV and BRV had significant effects on the latency of stage 3 seizures and above, NS: 25.8 ± 1.5, LEV: 35 ± 1.8, BRV: 37 ± 2) (*p* < 0.05) (Figure 10D), the number of animals with severe seizures (NS: 58%, LEV: 38%, BRV: 36%) (*p* < 0.05) (Figure 10E), and mortality (NS: 27%, LEV: 17%, BRV: 15%) (*p* < 0.05) (Figure 10F). The observations in these animal models demonstrated the differential effects of BRV and LEV on acute seizures and neuronal hyperexcitability.

## 4. Discussion

The principal findings presented in this study are as follows: First, cell exposure to brivaracetam (BRV), a bioavailable LEV derivative, resulted in a concentration-dependent inhibition of *I*_K(M)_; however, it mildly depressed the amplitude of *I*_K(DR)_ and *I*_h_. Second, the presence of this agent inhibited the peak amplitude of *I*_Na_ in a concentration-dependent manner together with a leftward shift in the steady-state inactivation curve of the current. Third, in the case of the inside-out current recordings, addition of BRV to the intracellular side of the excised patch enhanced the probability BK_Ca_ channels that would be open. Fourth, in the mHippoE-14 hippocampal neurons, BRV was effective in suppressing the amplitude of peak *I*_Na_ with minimal changes in the activation and inactivation time courses of the current. These observations thus suggest evidence that, besides being a high-affinity ligand for SV2A [9,15,39,41,42,43,44,45], BRV can perturb the ionic currents specified herein, hence disclosing a potential additional impact on the functional activities of different excitable cells.

The effective IC_50_ values needed for BRV-induced inhibition of the *I*_K(M)_ or peak *I*_Na_ observed in the GH_3_ cells were estimated to be 6.5 or 12.2 µM, respectively. In addition to a measurable reduction in the *I*_K(M)_ amplitude in response to long-lasting maintained depolarization, the activation time course of the current became slower in the presence of BRV. The BRV molecule can therefore reach the binding site once the *I*_K(M)_ channels are overly activated and reside in either the open state or in the open conformation. Additionallly, despite the decrease in peak *I*_Na_ combined with a lack of the overall IV relationship to the current, the steady-state inactivation curve of the current was found to shift along the voltage axis toward a hyperpolarized potential (about 9 mV), with no measurable perturbations on the estimated gating charge related to the conditioning potential versus the relative current amplitude. Consequently, the window *I*_Na_ [70] was expected to decrease in the presence of BRV. By extension, in the inside-out current recordings, the addition of BRV to the internal leaflet of the excised patch conceivably elevated the probability of BK_Ca_-channel openings, notwithstanding its inability to modify the single-channel amplitude. In the continued presence of BRV, subsequent application of either GAL-021 or PF1022A were detected to effectively attenuate increases in channel activity.

In previous recent pharmacokinetic studies on BRV, following intravenous administration of this agent, its plasma concentration was reported to range between 1 and 3 mg/L (i.e., 4.7 and 14.1 µM) [34,71,72]. Therefore, the ionic channels (i.e., M-type (*KCNQx*) K^+^, Na_V_ (*SCNx*), and BK_Ca_ (*KCNMA1*) channels) are a relevant target for the pharmacological actions of this drug and may virtually occur within the clinically therapeutic range although the detailed mechanism by which BRV interferes with actions on these types of ion channels still requires further detailed investigation.

It should be mentioned that glioma cells can functionally express the magnitude of *I*_Na_, which may be linked to the malignant transformation of neoplastic cells [73]. BRV was also previously reported to exert anti-neoplastic actions identified in glioma cells [40]. In keeping with previous observations [47], it was possible to determine that the presence of BRV can lead to a possible reduction in the amplitude of peak *I*_Na_ identified in GH_3_ or mHippoE-14 cells although the activation and inactivation time course of the current in response to the depolarizing command voltage remained unperturbed when the cells were exposed to BRV. As such, to what extent BRV-mediated changes in the magnitude of *I*_Na_ residing in glioma cells will participate in its anti-neoplastic actions remains to be established. Alternatively, BRV-induced relief of pain sensation as reported previously [1,2,39] could be partly explained by its inhibition of peak *I*_Na_ in sensory neurons.

It is worth noting that Levetiracetam was previously shown to decrease the amplitude of *I*_K(DR)_ when accompanied by an enhanced inactivation current time course [62]. However, in this study, the amplitude of *I*_K(DR)_ or *I*_h_ was mildly inhibited by adding BRV, and minimal changes in *I*_K(DR)_ inactivation in response to different levels of sustained depolarization were observed in its presence. It is conceivable, therefore, that in contrast to LEV, the *I*_K(DR)_ may not be an obligate target with which the BRV molecule can interfere. Moreover, the effects of BRV and LEV on the various types of ionic currents demonstrated herein could not be solely explained by their binding to the synaptic vesicle protein 2A (SV2A) in hippocampal neurons and pituitary cells [41,42,43,45,74], which was thought to be the case in both synaptic or endocrine vesicle exocytosis and neurotransmitter release, although they have been observed to be high-affinity SV2A ligands [9,15,39,43,44].

In the theoretical study, it was possible to mimic the BRV action on central neurons. It was noted that when cells were exposed to BRV, the simulated frequency of neuronal AP firing elicited in response to different depolarizing stimuli was obviously decreased owing to the changes in the conductance values of the different types of ionic currents referenced above, in combination with the reduced AP amplitude. It is conceivable, therefore, that its effects on neuronal APs occurring in vivo will be affected.

There were fundamental differences between the two animal models. In OD-1 model, we needed to anesthetize these animals first to stereotactically inject the OD-1 toxin and observed the seizure parameter 24 h later. There was thus no acute mortality in this group because of the initial anesthetic effect, which reduced the initial acute neuronal excitotoxicity. We evaluated the parameter severe seizures as an indicator of high neuronal excitotoxicity. For pilocarpine model, the acute excitotoxicity was prominent after initial intraperitoneal injection, thus it is not uncommon to lead to acute mortality following stage 4–5 seizure and status epilepticus. The latency to acute stage 3 seizures, number of rats with severe seizures and acute mortality were used to evaluate the high neuronal excitotoxicity. Therefore, the seizure parameters evaluated were different in both models.

The present evaluation of the effects of BRV versus LEV on acute seizure animal models further demonstrated the different ionic effects of BRV and LEV. Compared to LEV, sodium channel modulation of BRV, as demonstrated in the present study, explained its significant effect on OD-1, a unique sodium channel-agonism-based animal model of epilepsy and seizure. However, our previous study on LEV’s ionic mechanism did not reveal the underlying LEV mechanism for sodium channel modulation [62]. Nevertheless, both LEV and BRV had significant effects on the lithium-pilocarpine-induced seizure model, which was in line with previous observations regarding LEV’s effect on this model and the common SV2A mechanism of both medications in this model [75,76]. Furthermore, the various actions associated with the mechanism accounted for the finding that the response in the presence of BRV has been found to be several minutes faster than that with LEV in patients with photosensitive epilepsy [77], and BRV has been shown to be a useful treatment option in patients with epilepsy who have previously failed to respond to or tolerate LEV [48,78]. The unique ionic mechanism, in addition to the common SV2A modulation, justifies the role of BRV in rationale polytherapy for epileptic disorders, in terms of both efficacy and adverse events.

## 5. Conclusions

BRV’s multiple ionic mechanism in electrically excitable cells and a concerted effect on neuronal excitability underlies its therapeutic potential in clinical neuronal hyperexcitability disorders. 

## Figures and Tables

**Table 1:** Parametric values used for the modeling of hippocampal CA1 pyramidal neurons in an attempt to mimic the experimental results obtained in the control, where brivaracetam (BRV) was not present and during exposure to 10 µM BRV.

Symbol	Description	Value (in Control)	Value (in the Presence of 10 µM BRV)
C_m_	Membrane capacitance (pF)	1	1
g_Na_	Na^+^ current conductance (mS/cm^2^)	35	17.5
g_Ca_	Ca^2+^ current conductance (mS/cm^2^)	0.08	0.08
g_KDR_	Delayed-rectifier K^+^ current conductance (mS/cm^2^)	6.0	5.4
g_A_	A-type K^+^ current conductance (mS/cm^2^)	1.4	1.4
g_M_	M-type K^+^ current conductance (mS/cm^2^)	1	0.7
g_KCa_	Ca^2+^-activated K^+^ current conductance (mS/cm^2^)	10	20
g_h_	Hyperpolarization-activated cation current conductance (mS/cm^2^)	0.4	0.36

## Data Availability

The datasets used and/or analyzed during the current study are available from the corresponding author on reasonable request.
